# Interleukin-12-mediated expression of matrix metalloproteinases in human periodontal ligament fibroblasts involves in NF-κB activation

**DOI:** 10.1042/BSR20170973

**Published:** 2017-11-21

**Authors:** Li Miao, Shujun Zhan, Jiyan Liu

**Affiliations:** 1 Department of Stomatology, PLA Army General Hospital, Beijing 100700, China; 2 Department of Oncology, PLA Army General Hospital, Beijing 100700, China

**Keywords:** Interleukin-12, Matrix metalloproteinases, NF-kappaB, Periodontal ligament, Periodontitis

## Abstract

Interleukin-12 (IL-12) is a proinflammatory cytokine, and its increased level correlates with the severity of periodontitis. However, its role in the pathogenesis of tooth periapical lesions is controversial and has not been completely clarified. The present study aimed to investigate whether IL-12 affects the expression of matrix metalloproteinases (MMPs) and tissue inhibitors of metalloproteinases (TIMPs) in human periodontal ligament fibroblasts (hPDLFs). After treatment with IL-12 for different times, real-time PCR and Western blotting were used to determine the mRNA and protein levels of *MMP-1, MMP-2*, MMP-3, *MMP-9, MMP-13, TIMP-1*, and *TIMP-2*, respectively. ELISA was applied to measure MMPs and TIMPs secretion production. The results indicated that IL-12 significantly increased the mRNA and protein expression levels of *MMP-1, MMP-3*, and *MMP-13*, but down-regulated *MMP-2* and *MMP-9* mRNA and protein expression in the hPDLFs. Furthermore, IL-12 (10 ng/ml) enhanced the secreted protein production of MMP-1, MMP-3, and MMP-13, and conversely lowered MMP-2 and MMP-9 secretion levels. However, IL-12 treatment did not exert a significant effect on the mRNA and protein levels of *TIMP-1* and *TIMP-2* and their secreted production. Additionally, IL-12 increased the phosphorylated levels of IκBα and nuclear factor-κB P65 (NF-κB P65), and promoted NF-κB P65 subunit nuclear translocation. Pretreatment with NF-κB inhibitor not only attenuated IL-12-induced IκBα and NF-κB P65 phosphorylation and inhibited NF-κB P65 subunit into nucleus, but also antagonized IL-12-mediated MMP-1, MMP-2, MMP-3, MMP-9, and MMP-13 expression in the hPDLFs. These findings indicate that NF-κB-dependent activation is possibly indispensable for IL-12-mediated MMP expression in hPDLFs.

## Introduction

Periodontitis is a chronic inflammatory disease characterized by loss of the connective tissue and alveolar bone [1], and it is widely regarded as one of the most common diseases worldwide, with a prevalence of 15–20% [2]. Periodontal health requires a balance between tissue proteolytic enzymes such as matrix metalloproteinases (MMPs) and their inhibitors. Connective tissue destruction is essentially controlled by MMPs, which contributes to destruction of gingival tissue and alveolar bone surrounding the teeth [3]. MMPs are a group of zinc metalloendopeptidases that degrade extracellular matrix (ECM). Excessive production of MMPs leads to accelerated matrix degradation, which is associated with pathologic conditions such as periodontitis and apical periodontitis [4]. According to their target protein, MMPs are divided into six groups: collagenases (MMP-1 and MMP-13), gelatinases (MMP-2 and MMP-9), stromelysins (MMP-3), matrilysins, membrane-type MMPs, and others, such as the macrophage metalloelastase (MMP-12) [5]. Normally, MMP activities are tightly regulated by their interaction with the tissue inhibitors of MMPs (TIMPs) [6]. Thus, the factors that regulate MMPs/TIMPs synthesis and secretion may be important in the pathogenesis of chronic periodontitis.

Interleukin-12 (IL-12) is an important regulatory cytokine, involving both innate and adaptive immune responses [7]. IL-12 is mainly secreted by macrophages, monocytes, dendritic, and B cells in response to bacterial products and intracellular parasites [8]. Previous studies have indicated that the most important functions of IL-12 are to stimulate T and NK cells to produce interferon-γ (IFN-γ) and to promote the Th1 response [9,10], and it has been implicated in the pathogenesis of several diseases such as psoriasis [11], rheumatoid arthritis [12], and periodontitis [13]. Moreover, IL-12 has been reported to regulate osteoclast inhibitory peptide-1 (OIP-1) gene expression in CD4^+^ T cells [14].

In the periodontium, the most abundant cells are periodontal ligament fibroblasts (PDLFs), and they are responsible for the production of MMPs and TIMPs, and play a pivotal role in maintaining the functional integrity of the periodontal ECM [15], and have a principal role in the pathologic degradative processes of ECM in apical periodontitis [16]. Additionally, there is a higher level of IL-12 in gingival tissue and serum of patients with aggressive periodontitis [17,18]. However, little is known about the effects of IL-12 on the expression of MMPs in human PDLFs (hPDLFs) to date. Therefore, the aim of the present study was to determine the effects of IL-12 treatment on the mRNA and protein expression of MMP-1, MMP-2, MMP-3, MMP-9, MMP-13, TIMP-1, and TIMP-2 in the hPDLFs, and the mechanisms associated with IL-12-mediated MMP expression were also preliminarily explored.

## Materials and methods

### Antibodies and reagents

The primary antibodies recognizing MMP-1, MMP-2, MMP-3, MMP-9, MMP-13, TIMP-1, TIMP-2, and β-actin were purchased from Abcam Company (Cambridge, U.K.), and other antibodies, including anti-IκBα, anti-p-IκBα, anti-NF-κB P65 (anti-nuclear factor-κB P65), anti-p-NF-κB P65, and anti-Lamin A were obtained from Cell Signaling Technology (Danvers, MA, U.S.A.). Human recombinant IL-12 was purchased from R&D Systems (Minneapolis, MN, U.S.A.). MTT and pyrrolidine thiocarbamate (PDTC) were purchased from Sigma–Aldrich Company (St. Louis, MO, U.S.A.).

### Cell culture

hPDLFs were obtained from Jining Company (Shanghai, China), and cultured in Dulbeccos modified Eagles medium (DMEM) (Gibco, Grand Island, NY) supplemented with 10% FBS and penicillin (100 U/ml) and streptomycin (100 mg/ml) in a humidified atmosphere of 95% air and 5% CO_2_ at 37°C. When the cells grew into confluence, they were detached with 0.25% trypsin–EDTA and subcultured for the following experiments.

### MTT assay

hPDLFs at passage 5 (P5) seeded in 96-well plates at 5 ×10^3^ cells/well were treated with 0, 5, and 10 ng/ml of IL-12 for 12, 24, and 48 h, based on our preliminary experimental results (results not shown). Cell viability was then evaluated by MTT assay. Briefly, after incubation, the cells were washed and incubated with 0.5 mg/ml MTT solution in DMEM for 4 h at 37°C. Formazan crystals of viable cells were dissolved in 150 µl DMSO, and the absorbance value was measured at 570 nm with a microplate reader (Titertek, Pforzheim, Germany). Cell viability was expressed as percentages of control group.

### Real-time PCR analysis

After incubation with IL-12 (0, 5, and 10 ng/ml) for 12 and 24 h, total RNA of hPDLFs at P5 was extracted with TRIzol reagent (Invitrogen, Carlsbad, CA) according to the manufacturer’s instructions. One microgram of total RNA per sample was synthesized into cDNA with a reverse transcription kit (TaKaRa, Tokyo, Japan). Real-time PCR was performed by quantitative PCR System (ABI 7300) with FastStart Universal SYBR Green Master (Tiangen Biotech Co., Beijing, China) as follows: 95°C for 15 s followed by 35 cycles at 94°C for 5 s, and 60°C for 30 s. The specific primers used in the present study were designed and listed in Table 1. Glyceraldehyde-3-phosphate dehydrogenase (*GAPDH*) was used as an internal control, and gene expression levels were calculated using the ^−2ΔΔ*C*^_t_ method.

**Table 1:** Primers used for the real-time PCR

Gene	Forward	Reverse	Product size (bp)
*MMP-1*	5′-TGCTCATGCTTTTCAACCAG-3′	5′-GGTACATCAAAGCCCCGATA-3′	125
*MMP-2*	5′-CGACTCTTCGCACTCGACT-3′	5′-TCAGGATTCCCTGTTCGTTGA-3′	240
*MMP-3*	5′-GTCACCAGACCCATGGCA-3′	5′-GTCAGATGGAAGGGTCCCCA-3′	320
*MMP-9*	5′-GTAGCCTTCTTCCTTCCGAG-3′	5′-TCCCGGATATCTGTTGCCTG-3′	282
*MMP-13*	5′-CTGGAGTGATACTGATAAC-3′	5′-CACTAGGCATCAAGATATTACT-3′	119
*TIMP-1*	5′-AACATTCCTGGTCACCCAAC-3′	5′-GTTCCTCAACCCAAAGACCG-3′	165
*TIMP-2*	5′-GACAACCTCGTAGGTAAG-3′	5′-CTCTTCTAGACACCCGCAAT-3′	235
*GAPDH*	5′-TCCAGCGGTTACTCAAAC-3′	5′-GAGGACTCTAGTAGCCTGC-3′	275

### Western blot analysis

hPDLFs at passage 6 (P6) were incubated with 10 ng/ml of IL-12 for 48 h or pretreated with the NF-κB inhibitor PDTC (10 µM ) or quinazoline (10 µM) for 1 h, followed by treatment with IL-12 (10 ng/ml) for 6, 12, or 48 h, and cells were washed and lysed in RIPA lysis buffer (Beyotime, Haimen, China) supplemented with a protease inhibitor cocktail set (Roche, Germany), while the cytoplasmic and nuclear extracts from cells were prepared with NE-PER™ Nuclear and Cytoplasmic Extraction Reagents (Thermo Fisher Scientific, U.S.A.). The protein concentration was measured using a BCA protein assay kit (Beyotime), and equal amounts of protein extracts were separated on SDS/PAGE (8–12% gel), and electrotransferred on to PVDF membranes (Millipore, Bedford, MA). After being blocked with 5% skim milk in 1×TBST(TBS and 1% Tween 20), the membranes were incubated with corresponding primary antibodies (1:500 or 1:800 diluted) at 4°C overnight. After washing three times with TBST, the membranes were then incubated with appropriate horseradish peroxidase conjugated secondary antibodies (1:100 dilution) at room temperature for 1 h, followed by detection using ECL system (7SeaPharmTech, Shanghai, China). The β-actin and Lamin A were used as the loading control for total proteins and nuclear proteins, respectively.

### ELISA assay

hPDLFs at passage 7 (P7) were seeded in 12-well plates at 3 × 10^5^ cells/well with 1 ml of serum-free medium, and incubated with or without IL-12 (10 ng/ml) for 24 and 48 h, and the culture supernatants were collected. Subsequently, the secreted amounts of MMP-1, MMP-2, MMP-3, MMP-9, MMP-13, TIMP-1, and TIMP-2 proteins by hPDLFs were determined with commercial ELISA kits (R&D Systems, Minneapolis, MN) following the instructions of manufacturer. In brief, 100 µl of the standards, blanks, and supernatant samples were separately added into 96-well plates that were precoated with anti-human MMP-1, followed by incubation for 3 h at 37°C. After washing three times with PBS, biotinylated anti-human MMP-1 antibody (diluted 1:800) was pipetted into the wells and incubated for 1 h at room temperature, followed by washing three times with PBS to remove the unbound biotinylated antibody. HRP-conjugated streptavidin was then added into the wells for 1-h incubation at room temperature. After washing three times with PBS, the substrate tetramethylbenzidine (100 µl) was added into each well and incubated for 0.5 h in the dark room, and a stop solution provided with the ELISA kit was pipetted into each well. The absorbance values of each well were read on a microplate reader at 450 nm. The level of MMP-1 protein in the samples was obtained by comparison with the standard curve. Each sample was measured in duplicate. Additionally, the levels of MMP-2, MMP-3, MMP-9, MMP-13, TIMP-1, and TIMP-2 protein were determined in the same way as following the above procedures.

### Statistical analysis

Data were shown as mean ± S.E.M. Statistical analysis was performed by the two-tailed Student’s *t* test using the SPSS 11.0 software (IBM, Chicago, IL). The value of *P* <0.05 was considered to be bh statistically significant.

## Results

### Effect of IL-12 treatment on the viability of hPDLFs

**Figure 1:**
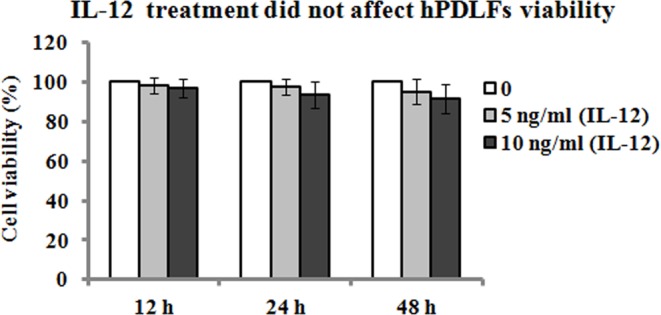
Effect of IL-12 on hPDLFs hPDLFs were treated with 0, 5, and 10 ng/ml of IL-12 for 12, 24, and 48 h, and cell viability was assessed by MTT assay. Data are expressed as percentage of cell viability relative to the control (0 ng/ml). Data represented as means ± S.E.M. (*n*=3).

The viability of hPDLFs was evaluated by MTT assay after IL-12 treatment for 12, 24, and 48 h. The results showed that 5 and 10 ng/ml of IL-12 did not result in a significant reduction in the viability of hPDLFs (Figure 1), and therefore, 5 and 10 ng/ml of IL-12 were considered to be non-cytotoxic, and were used in the following experiments.

### IL-12 promotes the expression of *MMP-1, MMP-3*, and *MMP-13*, and inhibited the expression of *MMP-2* and *MMP-9* in hPDLFs

**Figure 2:**
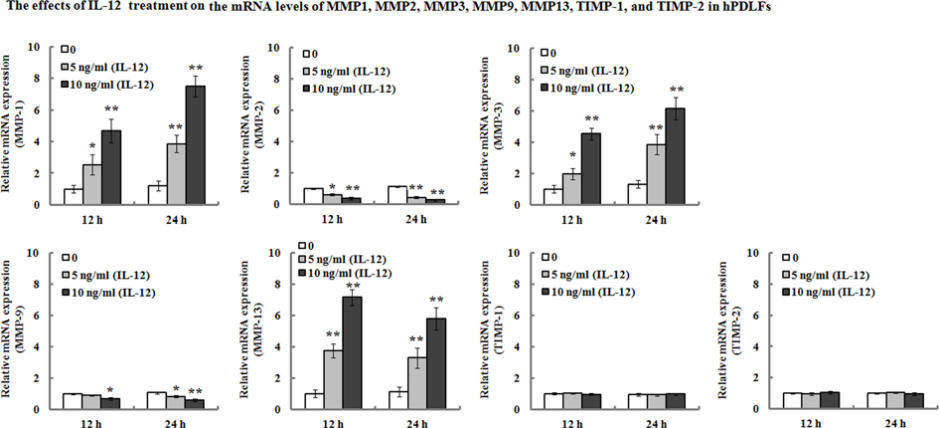
Effects of IL-12 on the mRNA levels of *MMP-1, MMP-2, MMP-3, MMP-9, MMP-13, TIMP-1*, and *TIMP-2* in hPDLFs hPDLFs were treated with 0, 5, and 10 ng/ml of IL-12 for 12 and 24 h, and then the mRNA expression levels of *MMP-1, MMP-2, MMP-3, MMP-9, MMP-13, TIMP-1*, and *TIMP-2* were determined by real-time PCR. Relative mRNA levels were presented as the ratios relative to untreated cells after normalization for their respective *GAPDH* mRNA expression. Data represented as means ± S.E.M. (*n*=3). *P<0.05 and **P<0.01, compared with the untreated group (0 ng/ml of IL-12).

hPDLFs were incubated with IL-12 (0, 5, and 10 ng/ml) for 12 and 24 h, and real-time PCR was used to determine the targetted gene expression. As shown in Figure 2, the results demonstrated that the mRNA expression levels of *MMP-1, MMP-3*, and *MMP-13* increased 2.54- (12 h), 3.87- (24 h), 1.98- (12 h), 3.84- (24 h), 3.75- (12 h), and 3.29- (24 h) folds, respectively, in the hPDLFs after exposure to 5 ng/ml of IL-12 for 12 and 24 h. When the cells were treated with 10 ng/ml of IL-12 for 12 and 24 h, their mRNA levels of *MMP-1, MMP-3*, and *MMP-13* increased 4.69- (12 h), 7.51- (24 h), 4.53- (12 h), 6.15- (24 h), 7.15- (12 h), and 5.78- (24 h) folds, respectively. On the contrary, the mRNA levels of *MMP-2* and *MMP-9* were significantly down-regulated, and their mRNA levels decreased by approximately 37% (12 h), 55% (24 h), 8% (12 h), and 18% (24 h), respectively, following the treatment of 5 ng/ml of IL-12 for 12 and 24 h. When the cells were treated with 10 ng/ml of IL-12 for 12 and 24 h, the mRNA levels of *MMP-2* and *MMP-9* decreased by approximately 61% (12 h), 72% (24 h), 31% (12 h), and 42% (24 h), respectively. However, *TIMP-1* and *TIMP-2* mRNA expression were not affected by IL-12 treatment.

**Figure 3:**
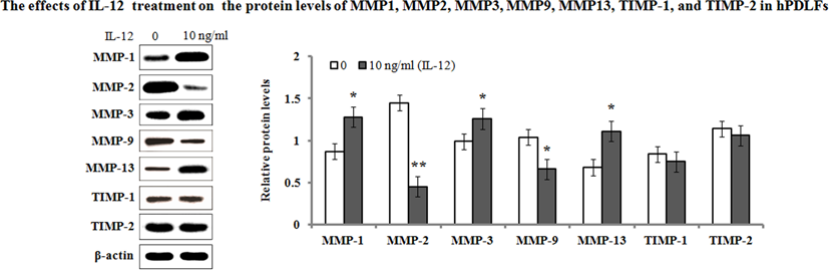
Effects of IL-12 on the protein levels of MMP-1, MMP-2, MMP-3, MMP-9, MMP-13, TIMP-1, and TIMP-2 After incubation with IL-12 (10 ng/ml) for 48 h, protein levels in the hPDLFs were analyzed with Western blotting. The presented Western blot image is a representative result of three independent experiments. Relative protein levels of MMP-1, MMP-2, MMP-3, MMP-9, MMP-13, TIMP-1, and TIMP-2 were normalized to β-actin signal bands. Data represented as means ± S.E.M. (*n*=3). *P<0.05 and **P<0.01, compared with the untreated group (0 ng/ml of IL-12).

**Figure 4:**
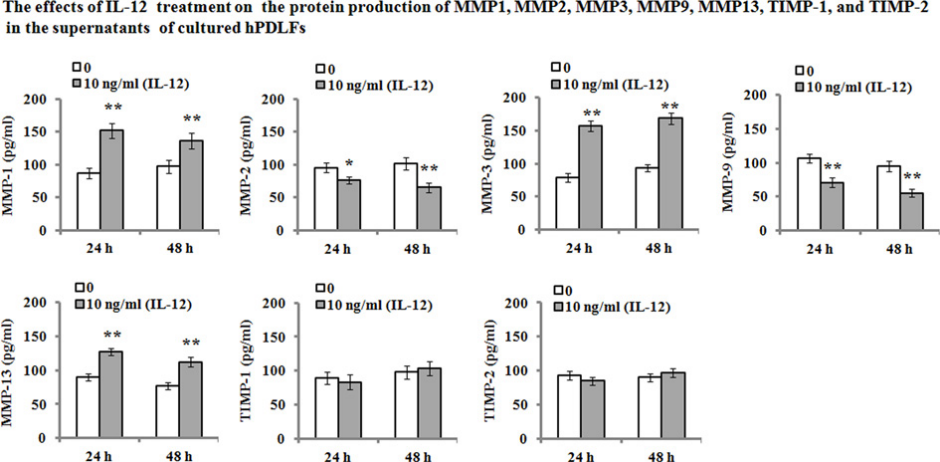
ELISA analysis for MMP-1, MMP-2, MMP-3, MMP-9, MMP-13, TIMP-1, and TIMP-2 hPDLFs were incubated in the absence or presence of 10 ng/ml of IL-12 for 24 and 48 h, and then protein production in the supernatants of cultured PDLFs was assayed by ELISA. Data are represented as means ± S.E.M. of three independent experiments. *P<0.05 and **P<0.01, compared with the untreated group (0 ng/ml of IL-12).

On the other hand, we also determined their protein levels of MMP-1, MMP-2, MMP-3, MMP-9, MMP-13, TIMP-1, and TIMP-2 in the hPDLFs after IL-12 treatment. The results of Western blot analyses showed that the protein expression levels exhibited a consistent trend with their mRNA levels (Figure 3). Additionally, ELISA analysis was performed to determine the protein production in the cultured hPDLF supernatants. As shown in Figure 4, the results demonstrated that IL-12 treatment resulted in a significant increase in the MMP-1 (from 86.29 and 97.04 pg/ml to 152.04 and 136.28 pg/ml, 24 and 48 h, respectively), MMP-3 (from 78.82 and 93.64 pg/ml to 156.41 and 168.27 pg/ml, 24 and 48 h, respectively), and MMP-13 (from 89.82 and 77.04 pg/ml to 127.57 and 112.33 pg/ml, 24 and 48 h, respectively) secretion when compared with the untreated groups. However, MMP-2 and MMP-9 production in the cultured medium were significantly decreased when compared with the untreated group. Their secretion was reduced from 95.45 and 102.09 pg/ml to 76.45 and 65.44 pg/ml in MMP-2 for 24 and 48 h, and from 106.48 and 94.73 pg/ml to 70.81 and 55.44 pg/ml in MMP-9 for 24 and 48 h, respectively. Additionally, there were no significant changes in TIMP-1 and TIMP-2 secretion in response to IL-12 treatment (*P*>0.05).

### IL-12-mediated MMP expression involves in NF-κB-dependent activation

**Figure 5:**
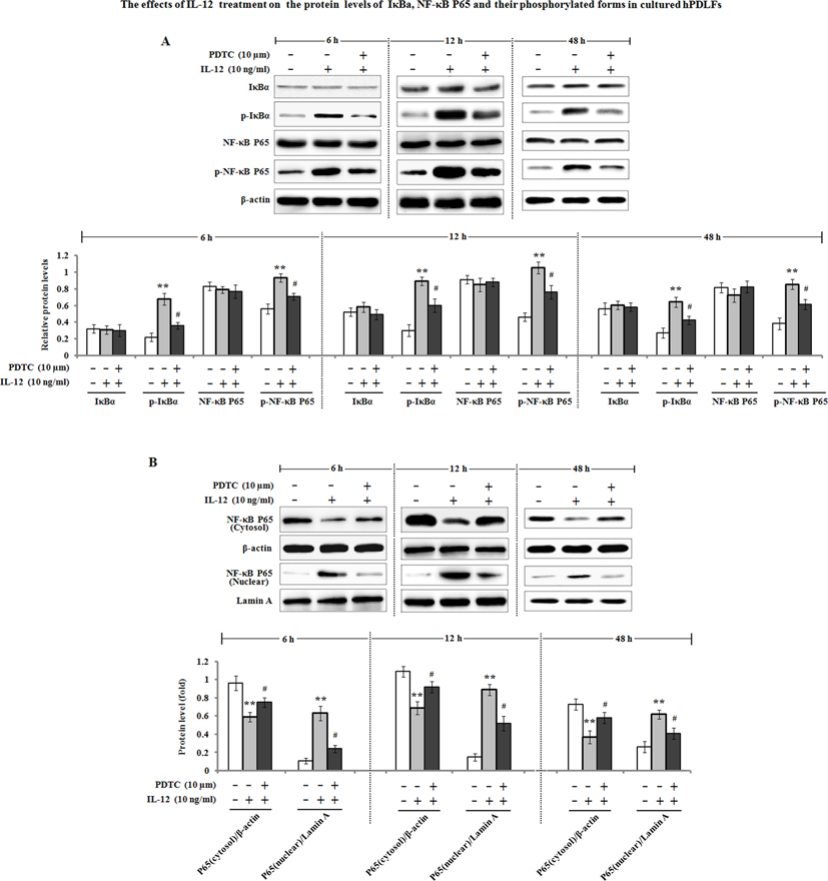
IL-12 induced the activation of NF-κB signaling pathway (**A**) hPDLFs were pretreated with 10 µM of PDTC for 1 h, followed by incubation with 10 ng/ml of IL-12 for, 6, 12, and 48 h, and total proteins were then extracted. The protein levels of IκBα, p-IκBα, NF-κB P65, and p-NF-κB P65 were subjected to Western blot analysis. β-actin served as the loading control. (**B**) hPDLFs were pretreated with 10 µM of PDTC for 1 h, and incubated with 10 ng/ml of IL-12 for 6, 12, and 48 h. The cytoplasmic and nuclear proteins were respectively prepared as described in the ‘Materials and methods’ section. Cytoplasmic and nuclear NF-κB P65 protein were analyzed with Western blotting. β-Actin and Lamin A expression served as the internal control of cytoplasm and nucleus, respectively. The fold increase in protein expression was normalized to β-actin or Lamin A. The presented Western blot image is a representative result of three independent experiments. Data are the means ± S.E.M. (*n*=3). **P<0.01 compared with the control (0 ng/ml of IL-12); ^#^*P*<0.05 compared with the IL-12 group.

**Figure 6:**
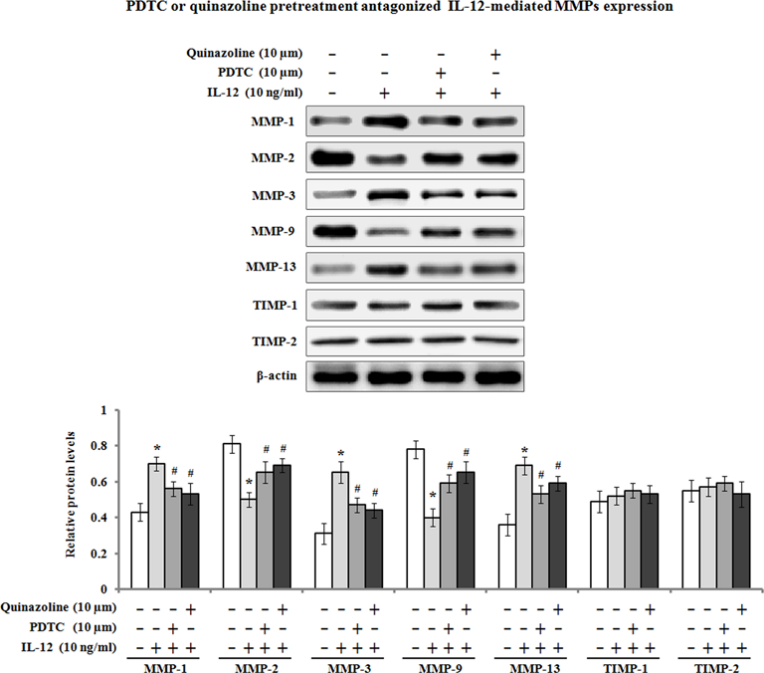
IL-12-mediated MMP expression involved in activation of NF-κB signaling pathway hPDLFs were pretreated with the inhibitor of NF-κB PDTC (10 µM) or quinazoline (10 µM) for 1 h, followed by treatment with IL-12 (10 ng/ml) for 48 h, and cellular total proteins were extracted. The protein expression levels of MMP-1, MMP-2, MMP-3, MMP-9, MMP-13, TIMP-1, and TIMP-2 were analyzed by Western blot analysis. The presented Western blot image is a representative result of three independent experiments. Data are the means ± S.E.M. (*n*=3). *P<0.05 compared with the control (0 ng/ml of IL-12); ^#^*P*<0.05 compared with the IL-12 group.

Previous studies have demonstrated that NF-κB can stimulate the expression of MMPs at transcriptional levels [19], and its activation is necessary for MMP-1 production by hPDLFs [20].Therefore, we determined the effects of IL-12 on IκBα, NF-κB P65 proteins and their phosphorylation levels (p-IκBα and p-NF-κB P65). The results showed that a significant increase of phosphorylated levels of IκBα and NF-κB P65 in the hPDLFs was observed after incubation with 10 ng/ml of IL-12 for 6, 12, and 48 h, but the protein levels of IκBα and NF-κB P65 remained unchanged (Figure 5A). Further investigation found that pretreatment with PDTC, an inhibitor of NF-κB pathway, markedly attenuated the phosphorylated forms of IκBα and NF-κB P65. Additionally, the nuclear translocation of NF-κB P65 subunit was investigated by Western blot analysis. As shown in Figure 5B, NF-κB P65 protein level in the cytoplasm was significantly decreased by IL-12 treatment, contrary to the up-regulation of NF-κB P65 protein level in the nucleus, suggesting that IL-12 treatment facilitated NF-κB P65 subunit nuclear translocation. Pretreatment with PDTC also antagonized IL-12-induced NF-κB P65 protein level in the cytoplasm and nucleus. On the other hand, we determined whether NF-κB activation was required for IL-12-dependent MMP regulation; pharmaceutical inhibitor of NF-κB pathway PDTC or quinazoline was used. The PDLFs were pre-incubated with 10 µM of PDTC or quinazoline for 1 h, followed by treatment with IL-12 for 48 h to examine the expression of MMP-1, MMP2, MMP-3, MMP-9, MMP-13, TIMP-1, and TIMP-2. As shown in Figure 6, the results of Western blot analysis exhibited that the enhancement of MMP-1, MMP-3, and MMP-13 was inhibited by PDTC or quinazoline pretreatment. Meanwhile, IL-12-induced MMP-2 and MMP-9 down-regulation was blocked by the inhibitor of NF-κB PDTC or quinazoline.

## Discussion

IL-12 is an important regulator of cellular immunity in both innate and adaptive immune response, and it increases the proliferation of T cells and NK cells, and stimulates the production of numerous immune effector molecules, in particular IFN-γ [21]. The expression of IL-12 has been found to be increased in periapical lesions after experimental pulp exposure, and macrophages and dendritic cells are probably responsible for IL-12 secretion [22]. Previous studies have implicated IL-12 in the pathogenesis of periodontal disease because of its effects on the expression of receptor activation of nuclear factor-κB ligand (RNAKL), a potent osteoclast-stimulating factor in human periodontal ligament cells [23]. In this study, we found that IL-12 was able to up-regulate the mRNA and protein expression of *MMP-1, MMP-3*, and *MMP-13* in hPDLFs, which contribute to tissue degradation in periapical areas. Additionally, we also found that the pretreatment on hPDLFs with an inhibitor of NF-κB pathway (PDTC or quinazoline) dramatically attenuated the increase of MMP-1, MMP-3, and MMP-13 protein expression, which suggests that IL-12-mediated MMP expression is possibly regulated through the activation of NF-κB pathway in hPDLFs.

MMP-1 is a key enzyme involved in degrading collagen types I and III, which are the most abundant components of the periodontal tissue matrix [24]. In healthy periodontal tissues, the level of MMP-1 is relatively low, which is thought to contribute to its physiological turnover [25]. However, the increase of MMP-1 protein induced by pulpitis or periapical periodontitis can lead to pathological processes, including ECM breakdown [26]. In the present study, the up-regulation of *MMP-1* mRNA and protein levels was observed after the PDLFs were exposed to IL-12, whereas TIMP-1 and TIMP-2 expression seemed to be unchanged after IL-12 treatment. Previous studies have shown the presence and immunolocalization of MMP-1 and TIMP-1 in human radicular cysts [27]. Our findings suggested an imbalance in MMP-1/TIMP-1 expression. MMP-1 activity is strictly regulated by TIMP-1. The balance between MMP-1 and TIMP-1 is a critical control point in connective tissue remodeling, and their imbalance may contribute to tissue destruction and progression of periapical lesions [28].

Additionally, the expression of MMP-3 in the IL-12 treatment group was significantly elevated when compared with the untreated group. MMP-3 is a broad-spectrum MMP and serves as a pivotal activator of latent MMPs, and it has been linked to tissue destruction associated with chronic inflammatory disease such as periodontitis [29]. In addition, MMP-3 is effective at degrading fibronectin, laminin, gelatins, proteoglycans, types IV and IX collagen [30]. In addition to its direct degradative activity, MMP-3 may also function indirectly by activating MMP-1 [31]. Several studies have demonstrated the co-ordinated expression and release of both MMP-1 and MMP-3 in human fibroblasts [32,33]. Accordingly, MMP-3 may play an important role in the overall regulation of the connective tissue degradation in pathologic conditions. In addition, it was reported that there is a substantial increase in the concentrations of MMP-3 and decrease of TIMP-1 in gingival crevicular fluid (GCF) in periodontal disease [34]. In the present study, the up-regulation of MMP-3 was observed after IL-12 treatment, which possibly contributes to progression of periodontal disease.

Additionally, MMP-13 (collagenase-3) expression was found in GCF samples from patients with chronic periodontitis, and its activity in these samples was significantly elevated [35]. Moreover, MMP-13 expression was also observed in gingival sulcular epithelium, macrophage-like cells, and gingival fibroblasts and plasma cells in chronic periodontitis [36]. MMP-13 not only is an enzyme responsible for bone resorption and cartilage destruction in rheumatoid arthritis and osteoarthritis [37], but also can degrade fibrillar type collagens, gelatin, basement membrane type IV collagen, fibronectin, tenascin, and proteoglycans [38]. These findings suggest that MMP-13 has important role in destruction of periodontal extracellular molecules, and is involved in the pathogenesis of periodontal disease [39]. It has been reported that proinflammatory cytokines such as IL-1, IL-6, IL-1β, and tumor necrosis factor-α can enhance MMP expression and production in human periodontal ligament cells [40]. In the present study, our findings showed that the expression levels of *MMP-13* mRNA and protein were up-regulated in hPDLFs after IL-12 treatment, which suggests that the increase of MMP-13 expression may be a crucial step in the deterioration of periodontal disease.

MMP-2 and MMP-9 belong to gelatinase-A (72 kDa) and gelatinase-B (96 kDa), respectively, and they act during the last phase of collagen degradation [41], and they are mainly responsible for the breakdown of type IV collagen and non-collagenous components of the ECM. In the present study, we investigated the effect of IL-12 treatment on the expression of MMP-2 and MMP-9 in hPDLFs. Unexpectedly, the results revealed that the mRNA and protein levels of MMP-2 and MMP-9 were significantly down-regulated by IL-12 treatment. IL-12 is an important cytokine in numerous immune functions in the initiation and regulation of cellular immune responses, and its production by macrophages or mast cells can enhance IL-1β expression and production [42]. Another study indicated that the mRNA and protein levels of MMP-1 were significantly increased by IL-1β stimulation [43]. In this study, it is likely that IL-12 treatment can enhance the promoter activity of *MMP-1, MMP-3*, and *MMP-13*, but inhibit the promoter activity of *MMP-2* and *MMP-9* through a direct or indirect regulatory effect in hPDLFs, which might result in different transcriptional effects on the expression of *MMPs*.

It has been reported that NF-κB activation is involved in the regulation of MMP expression [44], and as previously mentioned, its activation is necessary for MMP-1 production in hPDLFs [20,45]. In the present study, our data indicated that the levels of p-IκBα and p-NF-κB P65 were enhanced after IL-12 treatment on the hPDLFs. Furthermore, IL-12 treatment resulted in an increase of NF-κB P65 in the nucleus and decreased the level of NF-κB P65 protein in the cytoplasm, suggesting that IL-12 promoted the nuclear translocation of NF-κB P65 subunit in the hPDLFs. In addition, the pretreatment with PDTC, an inhibitor of NF-κB pathway, significantly antagonized IL-12-mediated MMP expression. These findings indicated that NF-κB activation is possibly required for IL-12-mediated MMP expression in hPDLFs. Similar results were obtained from previous studies [46].

In summary, our results indicate that IL-12 up-regulated the mRNA and protein expression of MMP-1, MMP-3, and MMP-13, but down-regulated that of MMP-2 and MMP-9 in hPDLFs. Additionally, IL-12 had no effect on the mRNA and protein levels of TIMP-1 and TIMP-2. Further investigation demonstrated that IL-12 treatment resulted in nuclear translocation of NF-κB P65 and NF-κB activation. Inhibition of NF-κB signal activation attenuated IL-12-mediated effects on MMP expression, suggesting that NF-κB signal pathway is probably required for IL-12-mediated MMP expression in hPDLFs. These findings also suggest that IL-12 may exacerbate periapical tissue destruction at least partly via regulating MMP expression during periapical inflammation.

## Abbreviations

DMEM: Dulbeccos modified Eagles medium
ECM: extracellular matrix
GCF: gingival crevicular fluid
hPDLF: human periodontal ligament fibroblast
HRP: horseradish peroxidase
IFN-γ: interferon-γ
IL-12: interleukin-12
MMP: matrix metalloproteinase
NF-κB: nuclear factor-κB
NK: natural killer
PDLF: periodontal ligament fibroblast
PDTC: pyrrolidine thiocarbamate
P5: passage 5
P65: nuclear factor-κB p65
TIMP: tissue inhibitor of metalloproteinase
